# Membrane Permeation of Psychedelic Tryptamines by Dynamic Simulations

**DOI:** 10.1021/acs.biochem.3c00598

**Published:** 2024-02-07

**Authors:** Vito F. Palmisano, Claudio Agnorelli, Andrea Fagiolini, David Erritzoe, David Nutt, Shirin Faraji, Juan J. Nogueira

**Affiliations:** † Department of Chemistry, Universidad Autonoma de Madrid, Madrid 28049, Spain; ‡ Theoretical Chemistry Group, Zernike Institute for Advanced Materials, University of Groningen, Groningen 9747 AG, The Netherlands; § Center for Psychedelic Research, Division of Psychiatry, Department of Brain Science, Imperial College of London, London SW7 2BX, U.K.; ∥ Unit of Psychiatry, Department of Molecular Medicine, University of Siena, Siena 53100, Italy; ⊥ IADCHEM, Institute for Advanced Research in Chemistry, Universidad Autonoma de Madrid, Madrid 28049, Spain

## Abstract

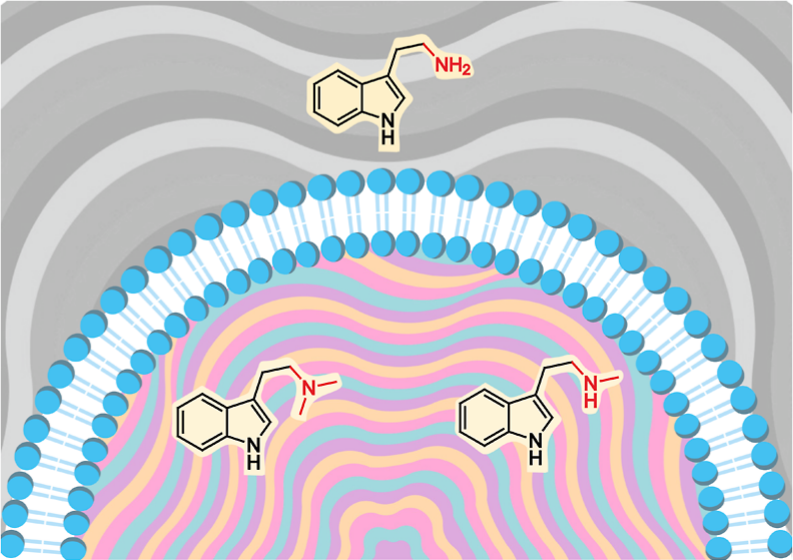

Renewed scientific interest in psychedelic compounds represents one of the most promising avenues for addressing the current burden of mental health disorders. Classic psychedelics are a group of compounds that exhibit structural similarities to the naturally occurring neurotransmitter serotonin (5-HT). Acting on the 5-HT type 2A receptors (HT_2A_Rs), psychedelics induce enduring neurophysiological changes that parallel their therapeutic psychological and behavioral effects. Recent preclinical evidence suggests that the ability of psychedelics to exert their action is determined by their ability to permeate the neuronal membrane to target a pool of intracellular 5-HT_2A_Rs. In this computational study, we employ classical molecular dynamics simulations and umbrella sampling techniques to investigate the permeation behavior of 12 selected tryptamines and to characterize the interactions that drive the process. We aim at elucidating the impact of N-alkylation, indole ring substitution and positional modifications, and protonation on their membrane permeability. Dimethylation of the primary amine group and the introduction of a methoxy group at position 5 exhibited an increase in permeability. Moreover, there is a significant influence of positional substitutions on the indole groups, and the protonation of the molecules substantially increases the energy barrier at the center of the bilayer, making the compounds highly impermeable. All the information extracted from the trends predicted by the simulations can be applied in future drug design projects to develop psychedelics with enhanced activity.

## Introduction

The prevalence of psychiatric diagnoses and the need for treatment in the population are rising steadily. In parallel, the efficacy of currently available treatments for debilitating conditions, such as depression, anxiety, addiction, and trauma, has proven to be insufficient, while the development of new pharmacological interventions to address the mental health epidemic has mainly produced insignificant variations of old drugs for decades.^1^ The recent comeback of psychedelics research represents one of the few promising areas of neuro-psychopharmacology, with the re-exploration of psychedelic-assisted psychotherapy and the development of new psychedelic analogues for the treatment of various mental health conditions.^2−4^

**Figure 1:**
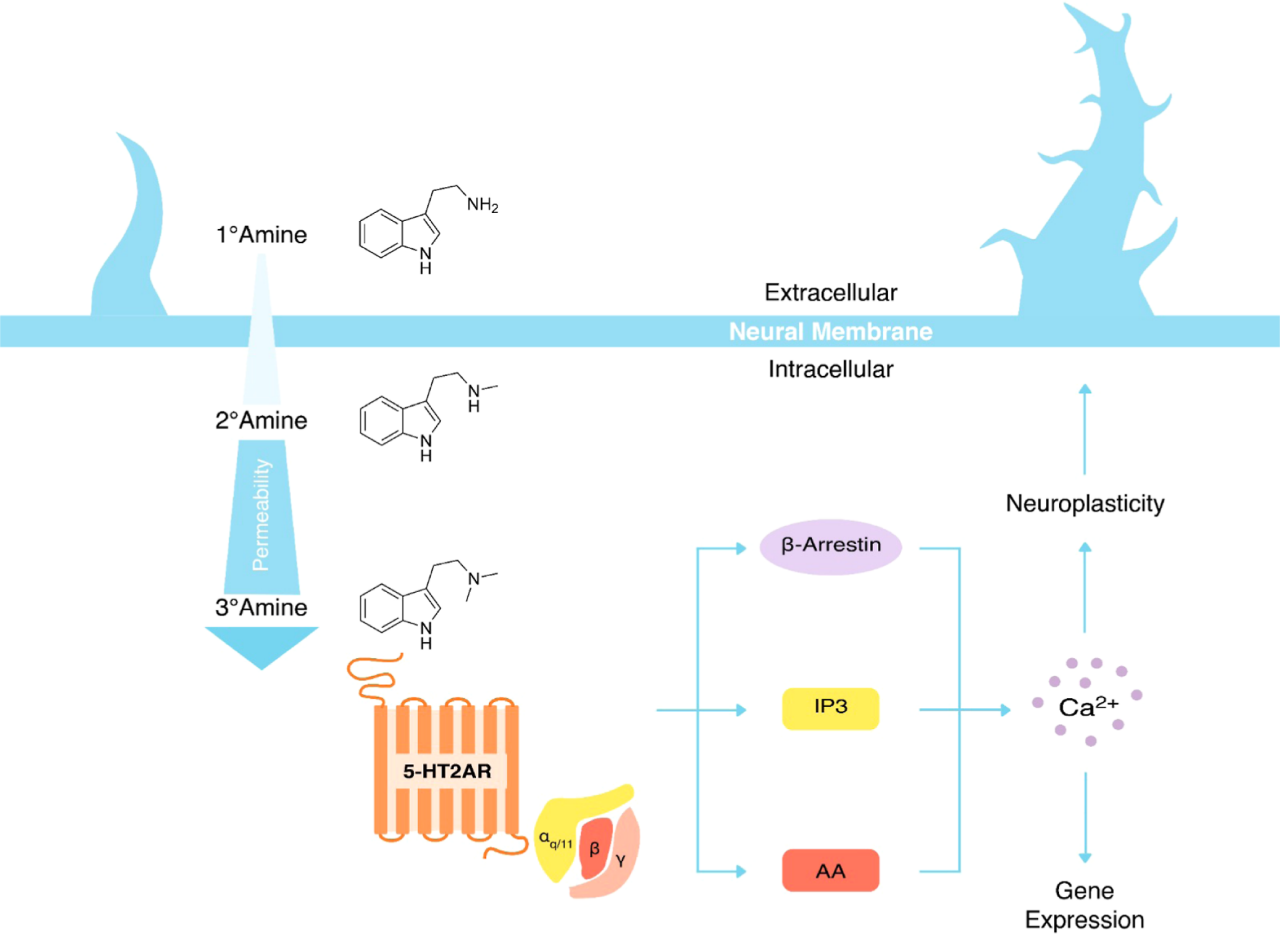
Model of the neuroplastic effects of classic psychedelics mediated by the intracellular 5-HT_2A_R postsynaptic molecular pathway. Modifying the primary amine group of tryptamines through alkylation influences their ability to penetrate the neural membrane, with only N-methylated and N,N-dimethylated tryptamines capable of entering the intracellular space. These compounds then bind to a pool of intracellular 5-HT_2A_Rs, triggering different pathways that induce changes in the structure and function of neurons.

Psychedelics are a class of psychoactive drugs comprising a variety of different compounds sharing structural similarities with the endogenous neurotransmitter serotonin or 5-HT.^5−7^ Classic psychedelics are defined by being agonists at the 5-HT type 2A receptor (5-HT_2A_R), and include molecules such as psilocybin, lysergic acid diethylamide (LSD), and *N*,*N*-dimethyltryptamine (*N*,*N*-DMT).^8^ In humans, the 5-HT_2A_R is densely expressed in cortical and neocortical regions involved in cognition, perception, sensorimotor gating, and mood.^9^ The activation of the 5-HT_2A_R by sufficient doses of classic psychedelics produces acute alterations in consciousness, accompanied by consistent brainwide neurophysiological changes, which are thought to mediate therapeutical effects.^10−12^ Animal studies have shown that psychedelics act at the apical dendrites of 5-HT_2A_R-expressing glutamatergic pyramidal neurons. Agonism at the 5-HT_2A_R triggers the mobilization of a cascade of kinases, leading to an increase of intracellular calcium (Ca^2+^), causing neuronal firing and expression of activity- and plasticity-related genes (Figure 1). As a result, there is a release of glutamate, especially in frontocortical regions, which triggers the release of neurotrophic factors, such as the brain-derived neurotrophic factor (BDNF), leading to changes in the structural and functional properties of neurons lasting several days following a single drug exposure.^13.17^

Importantly, the neuroplasticity-promoting effects of classic psychedelics have been found to underpin the alleviation of depression like behaviors in animal models.^14^ Similar to classic psychedelics, the endogenous ligand 5-HT and some non-psychoactive 5-HT_2A_R agonists also engage with 5-HT_2A_R-mediated pathways.^15^ However, the compound-specific variability in the relative neuroplastic effects that such molecules can produce is mechanistically poorly understood. While some 5-HT_2A_R agonists promote neuroplasticity without being psychoactive, classic psychedelics produce both subjective and neuroplastic effects, yet 5-HT and other non-psychoactive analogues have no such effects.^10−12^ Interestingly, the structural differences among some of these compounds amount to a few chemical substitutions from the molecular structure of the primary amine tryptamine. It has been proposed that different agonists can stabilize distinct active conformational receptor states, thus, interacting with different receptor residues to trigger specific subsets of signaling proteins coupled to the receptor, a phenomenon known as “biased agonism”.^16,17^

**Figure 2:**
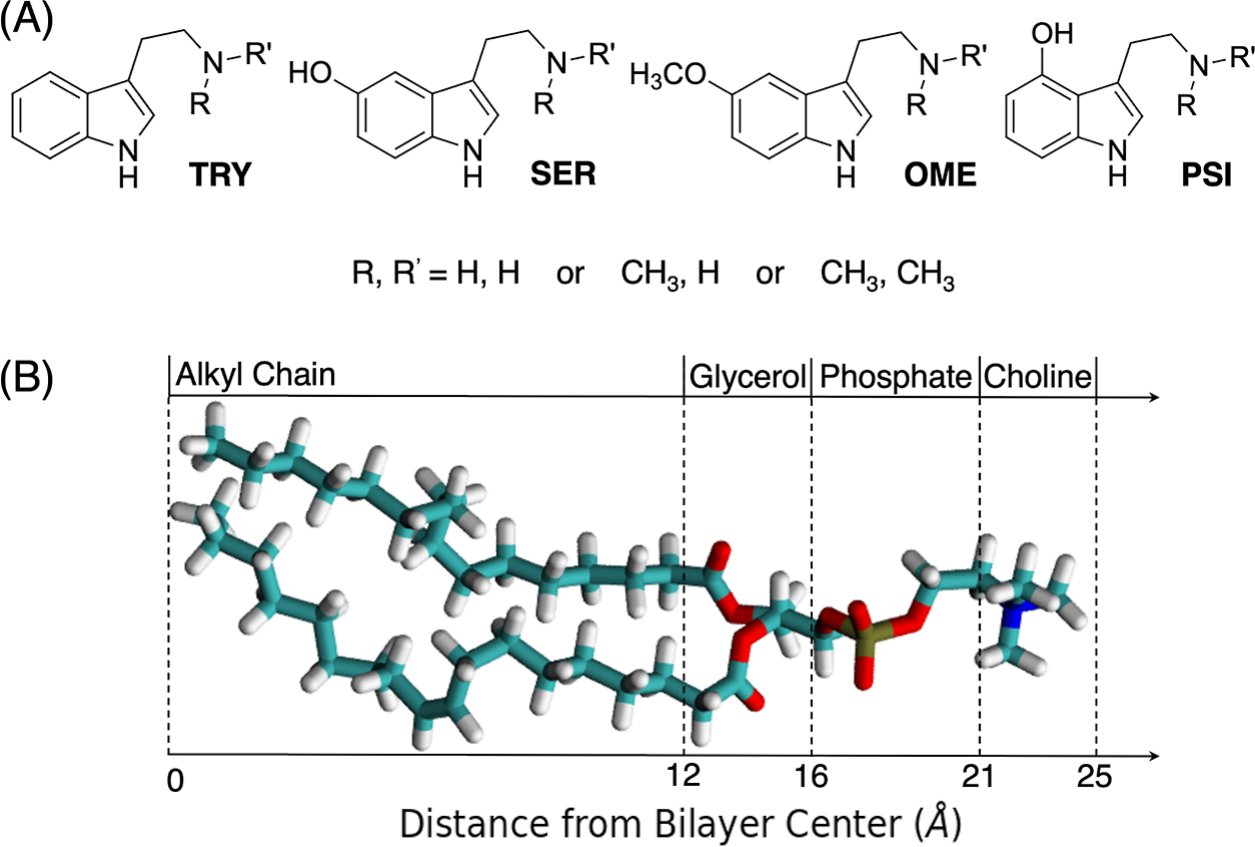
(A) Twelve molecules under investigation. (B) Licorice structure of a POPC lipid divided into its four main components: alkyl chain, glycerol, phosphate, and choline. Color code: C atoms are shown in cyan, hydrogen atoms in white, oxygen atoms in red, nitrogen atoms in blue, and phosphorus atoms in ochre.

Recently, Vargas et al.^18^ have observed another source of variability among the different 5-HT_2A_R agonists. First, they demonstrated that the activation of 5-HT_2A_R pools located intracellularly, rather than on the surface of the cellular membrane, is responsible for the neuroplastic effects induced by classic psychedelics. They also showed that when 5-HT is allowed to permeate the neuronal membrane via ectopic expression of the serotonin transporters, it can induce neuronal growth and elicit a head-twitch response (i.e., the behavioral correlation of psychedelic-induced subjective effects in rodents). The presence of intracellular clusters of the 5-HT_2A_R in various cell types has been demonstrated by a variety of previous in vitro experiments.^19^ Thus, in addition to biased agonism based on ligand–protein modulation, another phenomenon termed “location bias” may elucidate the divergent neuro-psychopharmacological characteristics of 5-HT_2A_R agonists. In addition, a significant positive correlation was found between the neuroplasticity-promoting properties and the lipophilicity of different 5-HT_2A_R agonists.^18^ Because of this, it has been suggested that a molecule’s ability to diffuse through the neuronal plasmatic membrane and interact with intracellularly localized receptors, thereby activating cellular pathways that are relevant for therapeutic effects, would depend on its polarity, which is established by particular chemical substitutions. In this context, it becomes crucial to establish a systematic approach that can predict the changes in the permeability of a compound resulting from chemical substitutions. Only one study has investigated the permeation of tryptamines into a 1-palmitoyl-2-oleoyl-*sn*-glycero-3-phosphocholine (POPC)/POPS membrane both in their protonated and neutral form.^20^ This study emphasized that protonated compounds face high energy barriers in the center of the membrane, while the permeability of neutral compounds is modulated by the substituents on the indole ring, which affect the compound’s polarity. In the present study, we went beyond previous computational research by performing a deeper analysis of the different structural factors that affect the membrane permeation of psychedelic tryptamines. Specifically, we employed classical molecular dynamics (MD) and umbrella sampling (US) techniques to investigate the permeation behavior of 12 tryptamines in their neutral form and two protonated analogues. These specific tryptamines were selected with the aim of unveiling separately the influence of N-alkylation, indole ring substitution, and position of substituents on their permeability across membranes. The set of compounds included tryptamine (TRY), serotonin (SER), 5-methoxy-tryptamine (OME), and 4-hydroxy-tryptamine (PSI), as well as their corresponding *N*-methyl and *N*,*N*-dimethyl substitutions (see Figure 2A). These compounds were modeled to permeate a model membrane formed by POPC lipid molecules (Figure 2B).

Furthermore, based on the symmetrically calculated potential of mean force (PMF), the logarithm of the effective permeability coefficient (log *P*_eff_) values obtained for all substituted tryptamines align well with the findings of Vargas et al. (2023). Our findings underscore the essential requirement of the neutral form of tryptamines for effective permeation through the lipid bilayer. Furthermore, we note that both alkylation and methoxy substitution exert the most pronounced influence on the overall polarity of the compound, and we could predict the behavior of PSI compounds by examining the impact of substituting indole positions from 5 to 4. This substitution enhances the permeability of PSI compounds compared with their positional isomers, SER, in both unsubstituted and alkylated forms. Those result in the most significant alterations in permeation characteristics.

## Computational Details

### Initial Structures

**Figure 3:**
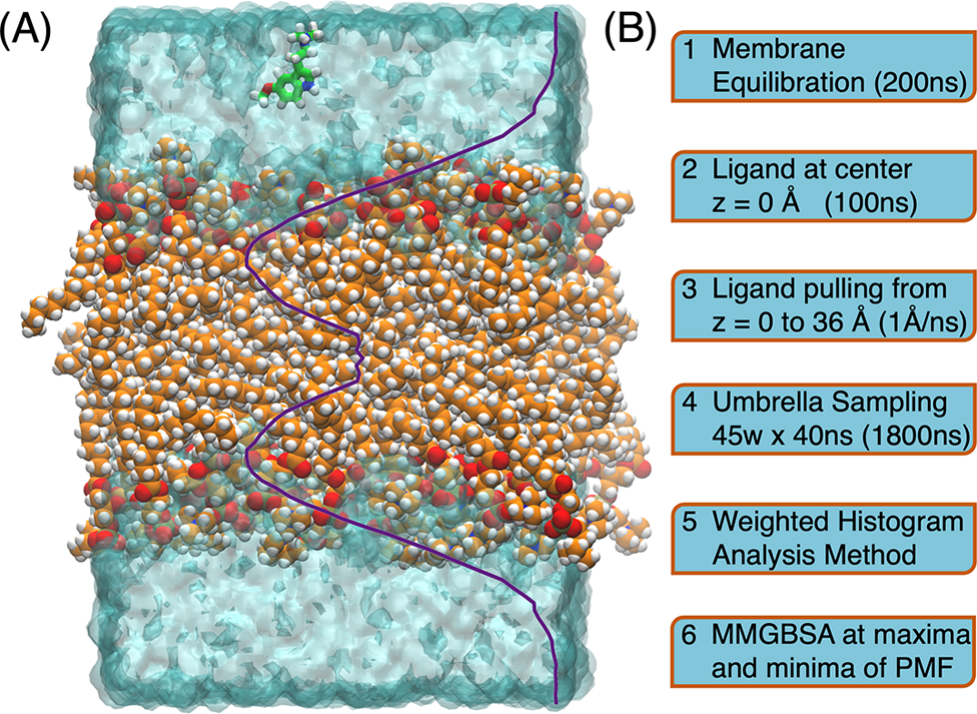
(A) Representation of *N*,*N*-OME (green) in bulk water (cyan) and a POPC lipid bilayer (orange). (B) Computational protocol followed for all the ligands under investigation.

For this study, a total of 12 tryptamines were selected: TRY, SER, OME, PSI, and their corresponding *N*-methyl and *N*,*N*-dimethyl substitutions (Figure 2A). Although the p*K*_a_ values of these compounds range from 8.6 to 10,^21^ indicating their tendency to be predominantly protonated under physiological conditions, this study specifically focused on the investigation of the neutral species, which can permeate the membrane and become ionized again in the cytosol. Previous research also has demonstrated that the charged analogues have the ability to partition into the lipid bilayer, but they face a significant energy barrier, making it more likely for the neutral species to cross the bilayer.^20,22^ The ligands were built using IQmol Molecular Viewer,^23^ and their geometries were optimized at the B3LYP/cc-pVDZ level of theory.^24^ The lipid bilayer, on the other hand, was constructed using the CHARMM-GUI Bilayer Builder.^25^ A total of 100 POPC molecules (Figure 2B) were distributed in two layers and solvated in a rectangular box with an aqueous solvent and NaCl at a concentration of 0.15 mol L^–1^. The choice of a homogeneous POPC membrane was inspired by a computational study focused on the neuronal plasma membrane,^26^ which merged findings from various lipidomics investigations of neurons and brain tissues. While PC is the predominant headgroup in the human membranes and contributes to its zwitterionic nature, it is important to highlight that the permeation of compounds may still be influenced by the presence of other lipids or cholesterol. These potential effects must be investigated in future studies. The dimensions of the box were as follows: *x* = 58 Å, *y* = 58 Å, and *z* = 86 Å. The potential parameters for the lipids and water were taken from the Lipid17 and TIP3P force fields,^27,28^ respectively, and Lennard-Jones parameters for ions were adopted from Joung and Cheatham.^29^ For the ligands, the restricted electrostatic potential charges^30^ were computed by the Merz–Singh–Kollman scheme at HF/6-31G* level with the Gaussian09 software^31^ to ensure a precise description of the neutral and ionized form, and the rest of parameters were taken from GAFF.^32^ The system is composed of 100 POPC molecules, representing the lipid bilayer, along with 5000 water molecules, 22 Na^+^Cl^–^ ions, and the ligand of interest, resulting in a total system size of approximately 28,500 atoms (Figure 3A), comparable in size with similar studies.

### Classical MD

MD simulations of the solvated membrane were performed using the CUDA version of the AMBER20 package.^33^ Initially, an energy minimization was conducted for 5000 steps with the steepest descent method, followed by an additional 5000 steps using the conjugate gradient method. Then, positional restraints on the membrane were gradually released, ranging from 10 to 1 kcal mol^–1^ Å^–2^, while the system was heated in the *NVT* ensemble from 0 to 303.15 K utilizing the Langevin thermostat with a relaxation time of 1.0 ps for a total simulation time of 1200 ps and 2 fs time step. To achieve the desired density, an equilibration phase was carried out in the *NPT* ensemble with a Monte Carlo barostat and semi-isotropic pressure scaling and a Langevin thermostat with a relaxation time of 1 ps for 500 ps with a time step of 2 fs. Following the equilibration phase, a production run of 200 ns was conducted to allow for further equilibration of the structure of the lipid bilayer prior to the insertion of the tryptamines. The ligands were then inserted into the model at the center of the membrane (*z* = 0 Å). To further stabilize the membrane in the presence of the ligand, an energy minimization was performed for 5000 steps with the steepest descent method, followed by an additional 5000 steps using the conjugate gradient method. The membrane and the ligand were subjected to positional restraints of 10 kcal mol^–1^ Å^–2^ while being heated in the *NVT* ensemble from 0 to 303.15 K with the Langevin thermostat with a relaxation time of 1 ps for a total simulation time of 1200 ps and 2 fs time step. Then, an *NPT* simulation was evolved with a Monte Carlo barostat and semi-isotropic pressure scaling and a Langevin thermostat with a relaxation time of 1 ps for 500 ps with a time step of 2 fs. Subsequently, a single harmonic restraint of 2.5 kcal mol^–1^ Å^–2^ was applied to maintain the distance between the center of mass (COM) of the POPC bilayer and the initial position of the ligand at *z* = 0 Å during a production run of 100 ns. A pulling run was conducted to gradually diffuse the ligand from the membrane center (*z* = 0 Å) to the bulk water phase (*z* = 36 Å). The pulling rate employed was 1 Å/ns. This pulling process was performed to obtain the initial geometries for subsequent US simulations, which are described later. Pulling the ligand from the center of the membrane toward the water phase is beneficial for achieving faster convergence of the PMF compared to pulling from the water phase into the membrane.^34^ During the entire computational protocol, the SHAKE algorithm was employed to constrain the bond lengths involving hydrogen atoms, while the cutoff radius for nonbonded interaction was set at 10 Å.^35,36^ Electrostatic interactions were computed using the particle-mesh Ewald method with a grid spacing of 1 Å.^37,38^

### Free Energy Calculations

US was employed to explore the diffusion pathway from the center of the bilayer to the bulk solvent. In this approach, a single harmonic restraint with a force constant of 2.5 kcal mol^–1^ Å^–2^ was applied to restrain the value of the reaction coordinate, which was defined as the *z*-axis component of the distance between the COM of the POPC bilayer and the COM of the compounds. The reaction coordinate was divided into 45 windows spaced at intervals of 0.8 Å along the *z*-direction from the center of the membrane (*z* = 0 Å) to the water phase (*z* = 36 Å). In each window, an MD trajectory was evolved for 40 ns and the analyses were performed on the full 40 ns of each simulation. The weighted histogram analysis method was used to calculate the PMF along the reaction coordinate for each compound based on the data obtained from each US simulation.^39,40^ The free energy profile along a coordinate *z*, *W*(*z*), or PMF and the local diffusivity coefficient, *D*(*z*), can be related to the effective resistivity, *R*_eff_, and effective permeability, *P*_eff_, through the following equation
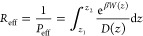
1where, β represents the reciprocal of the product of Boltzmann’s constant and temperature (1/*k*_b_*T*), and z serves as a reaction coordinate characterizing the solute’s position along the lipid bilayer.^41,42^ Both terms *W*(*z*) and *D*(*z*) were determined from the US simulations. To characterize the underlying intermolecular interactions responsible for the diffusion of the compounds, the binding free energy for each minimum and maximum along the PMFs was determined using the molecular mechanics generalized Born surface area (MMGBSA) approach.^43^ For each MMGBSA calculation, 500 frames were chosen, and the total energy (Δ*G*_tot_) was decomposed into van der Waals (Δ*G*_vdW_) and electrostatic (Δ*G*_el_) interactions between the ligand and the lipid bilayer. The electrostatic term represents the Coulombic interaction between the atomic charges of the different atoms, while the vdW term accounts for all the non-electrostatic interactions, including Pauli repulsion, induction, and dispersion interactions. Additionally, the interaction between the ligands and the implicit solvent (Δ*G*_gb_) is decomposed into polar (Δ*G*_pol_) and nonpolar (Δ*G*_np_) terms. This method offers valuable insights into the nature of the interactions involved, allowing for a rational analysis of the position and depth of the minima with a low computational cost.

## Results and Discussion

### Overall Permeation

Table 1 presents the log *P*_eff_ values obtained through PMF analysis (see Supporting Information for an analysis of the PMF convergence) for each of the compounds studied. A positive log *P*_eff_ indicates that the compound is permeable, while a negative log *P*_eff_ value indicated impermeability. Notably, the dimethylated compounds display positive permeability, and among the N-methylated variants, all except *N*-SER exhibit positive permeability as well. Within the unmethylated compound group, OME demonstrates the highest permeability, followed by PSI, TRY, and SER in respective order. The two protonated species, TRY^+^ and *N*,*N*-TRY^+^, are highly impermeable.

**Table 1:** Log *P*_eff_ of All the Molecules under Investigation

compound	log *P*_eff_
SER	–2.25
*N*-SER	–1.95
*N*,*N*-SER	1.31
PSI	–0.66
*N*-PSI	0.79
*N*,*N*-PSI	1.51
TRY	–1.06
TRY^+^	–2.65
*N*-TRY	1.20
*N*,*N*-TRY	1.59
*N*,*N*-TRY^+^	–6.30
OME	0.51
*N*-OME	1.28
*N*,*N*-OME	1.53

**Figure 4:**
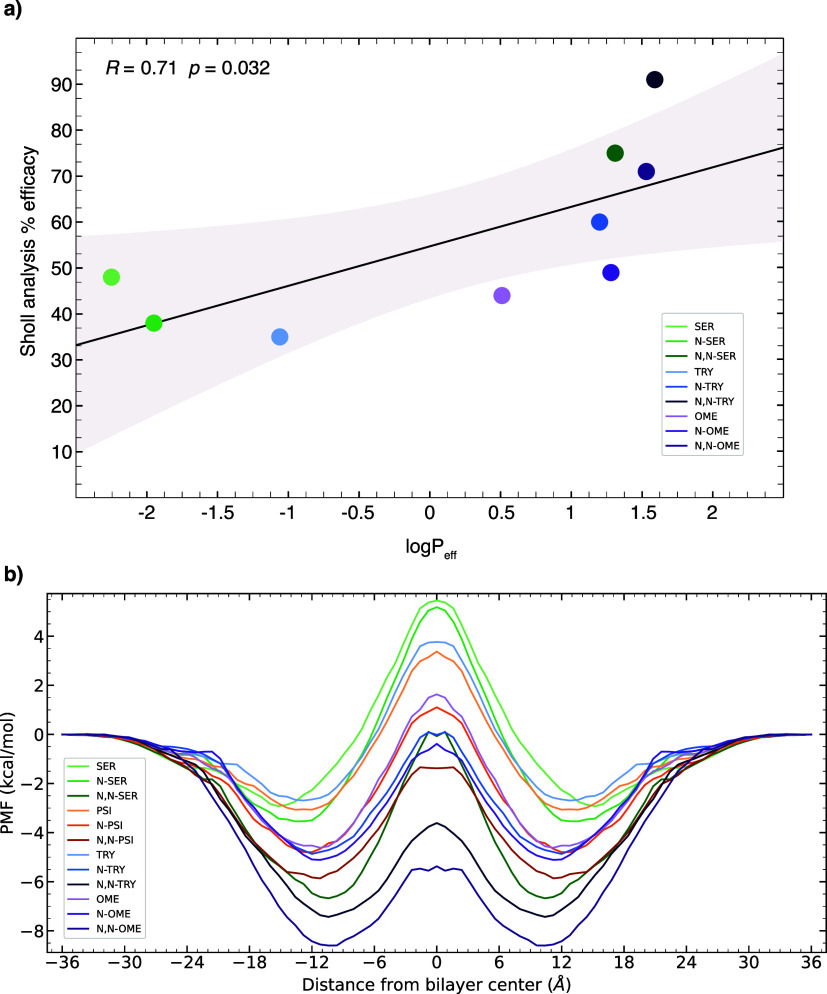
(A) Log *P*_eff_ vs the experimental dendritogenesis efficacy of the nine drugs studied in Vargas et al. *R* corresponds to the Pearson correlation coefficient and p corresponds to the statistical significance at α = 0.05. (B) Symmetric PMF of all the molecules under investigation.

The log *P*_eff_ values, calculated from the PMF analysis, were then plotted against the dendritogenesis efficacy data as experimentally obtained by Vargas et al.,^18^ as shown in Figure 4A, with the exception of the PSI compounds for which experimental data were not accessible. The log *P*_eff_ values also closely align with those computed by Vargas et al. using the Molispiration milogP predictor. Interestingly, a notable difference in log *P*_eff_ is observed between the SER and PSI compounds despite their similar amphipathic character. This intriguing disparity will be explored further in the subsequent section. All the compounds permeate from the bulk water (*z* = 36 Å) phase to the membrane’s head groups (*z* = 25 Å), as illustrated in Figure 4B, in a barrierless manner. Additionally, these compounds exhibit energy minima within a broad range of reaction coordinate values, situated approximately 14 to 10 Å away from the membrane’s center, depending on the ligand. This behavior can be attributed to their amphipathic nature, enabling interactions with both the head groups and the hydrophobic tail regions of the membrane. As will be discussed below, the nature of the compound will determine the strength of the different interactions with tails and heads and, therefore, the position of the free energy minima. Compared to bulk, this location is favored by ≈ 2 kcal/mol for the most shallowest minima attributed to TRY to ≈8 kcal/mol for *N*,*N*-OME. Beyond this point, all drugs encounter an energy barrier with respect to the minimum to pass through the center of the bilayer (*z* = 0 Å), which holds particular relevance for their permeability characteristics. In the following sections, different subsets of compounds are chosen to specifically analyze and discuss the impact of individual substitutions in greater detail.

### N-alkylation

**Figure 5:**
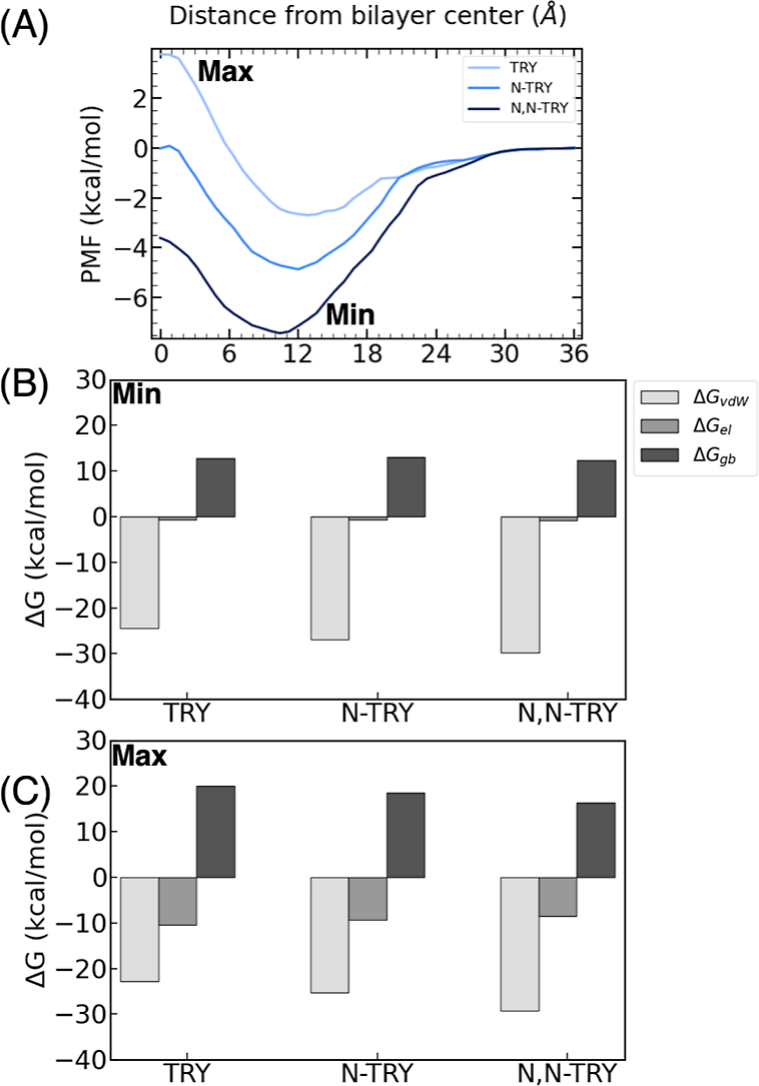
(A) PMF for TRY, *N*-TRY, and *N*,*N*-TRY. Decomposition of the energy into van der Waals (Δ*G*_vdw_), electrostatic (Δ*G*_el_), and implicit generalized Born solvation, (Δ*G*_gb_), in the (B) minima and (C) maxima of the compounds.

Our analysis will primarily focus on the alkylation of the primary amine group, which represents the most polar region within this class of compounds. This particular part of the molecule plays a crucial role in determining the membrane permeation efficiency. To investigate this, we compare the permeation behavior of TRY with its *N*-methyl and *N*,*N*-dimethyl analogues (Figure 5A).

The observed variance in the minimum position signifies the distinction in the hydrophobic properties among the three compounds. Specifically, the monoamine compound is situated in closer proximity to the membrane’s head groups (*z* = 13.6 Å), followed by *N*-TRY (*z* = 12.0 Å) and *N*,*N*-TRY (*z* = 10.4 Å). The positioning of these minima is related to the amphiphilic nature of these compounds, with a discernible decrease in polarity from TRY to *N*,*N*-TRY. Furthermore, it is noteworthy that the depth of the minimum energy increases when transitioning from TRY to *N*,*N*-TRY. This trend is also evident when comparing the unmethylated, monomethylated, and dimethylated variants of SER, TRY, and OME. The end-state free energy calculation, MMGBSA, shows an increase in Δ*G*_vdW_ going from the primary to the tertiary amine, which can be attributed to an increase in hydrophobic interactions with the lipid tail, and a decrease in electrostatic interactions, corresponding to a larger distance to the head groups (Figure 5B). The solvation energy contribution, Δ*G*_gb_, is slightly more unfavorable for TRY, followed by *N*-TRY and *N*,*N*-TRY, respectively. As expected, the solvent molecules exhibit stronger interactions with TRY when the ligand is situated within the bulk solvation environment since primary amines have two hydrogen atoms available for hydrogen bonding. Consequently, as the ligand transitions from an aqueous phase to a membrane environment, the energy penalty for the desolvation process is more significant for TRY compared to that for the substituted ligands. As we progress toward the center of the membrane, while the electrostatic and solvent interactions are comparable among the three compounds (see Figure 5C), the dimethylated compound continues to exhibit more favorable vdW interactions, meaning a stronger hydrophobic interaction due to increased methylation of the monoamine. Consequently, it experiences a lower energy barrier, which enhances its permeation ability.

### Indole Substitution

**Figure 6:**
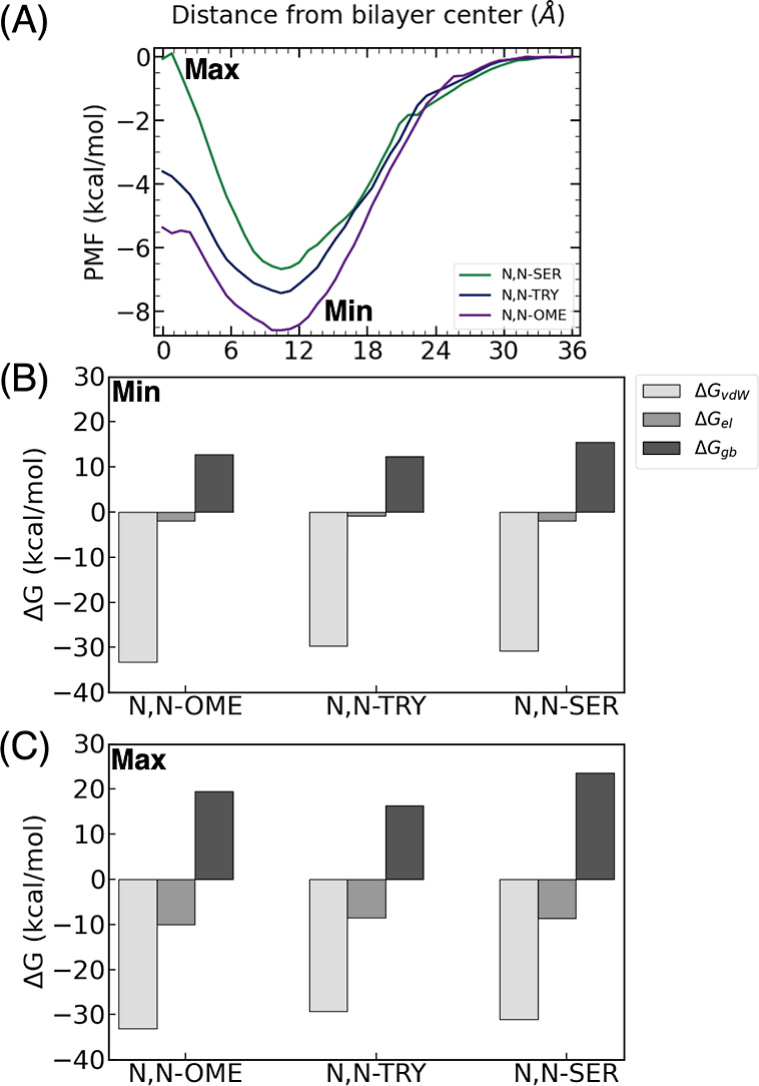
(A) PMF for *N*,*N*-OME, *N*,*N*-TRY, and *N*,*N*-SER. Decomposition of the energy into van der Waals (Δ*G*_vdw_), electrostatic (Δ*G*_el_), and implicit generalized Born solvation, (Δ*G*_gb_), in the (B) minima and (C) maxima of the compounds.

To investigate the impact of indole substitution, we selected three compounds: *N*,*N*-TRY, *N*,*N*-OME, and *N*,*N*-SER. The choice of these compounds was based on their shared substitution at position 5, with OME featuring a –OCH_3_ group and SER featuring a –OH group (Figure 6A).

Once again, it is observed that all of the compounds undergo diffusion from the bulk water phase to the headgroups without any energy cost. The minima for all compounds are located approximately 10.4 Å from the bilayer center, consistent with the trend observed in the previous section for the methylated compounds. The indole substitution does not alter the position of the minimum as the dimethylated amine group interacts strongly with the lipid tails, as discussed in the previous section. This suggests that the amine substitution has a greater influence on the position of the minimum than indole substitution. However, the indole substitution does affect the depth of the minimum. Notably, the interaction with the membrane is more favorable in the following order: *N*,*N*-OME > *N*,*N*-TRY > *N*,*N*-SER.

**Figure 7:**
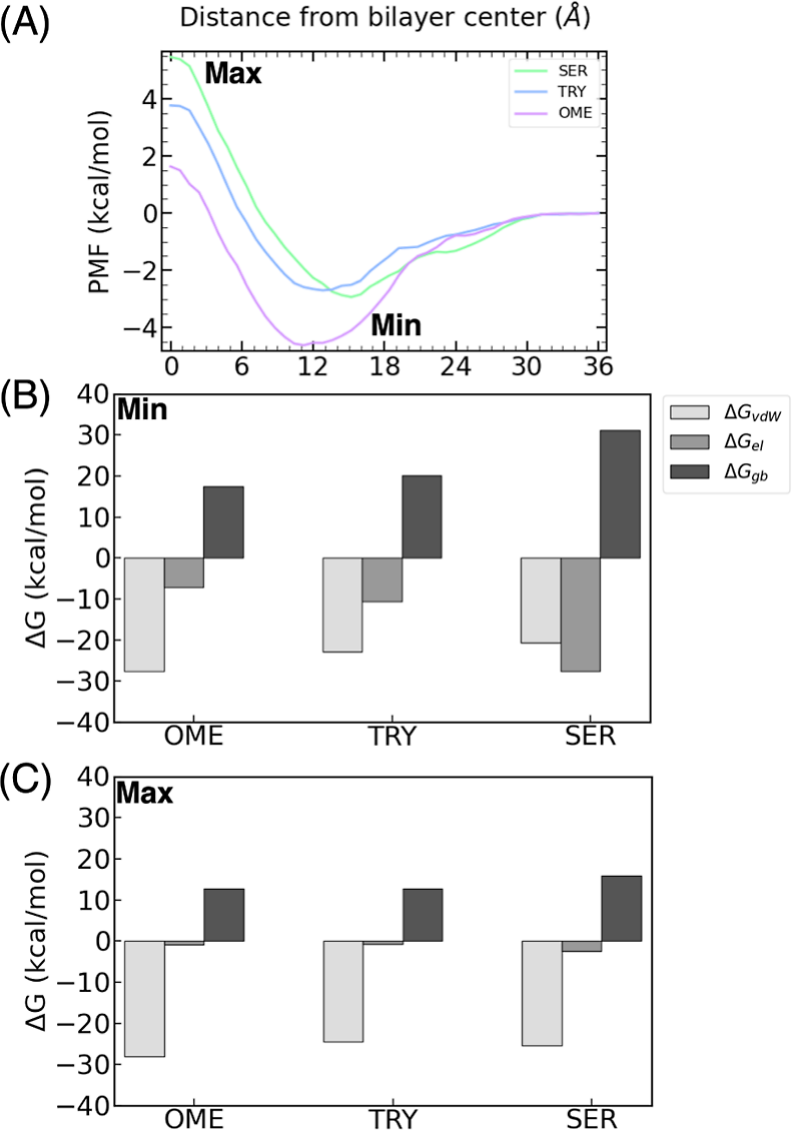
(A) PMF for OME, TRY, and SER. Decomposition of the energy into van der Waals (Δ*G*_vdw_), electrostatic (Δ*G*_el_), and implicit generalized Born solvation, (Δ*G*_gb_), in the (B) minima and (C) maxima of the compounds.

The observed trend can be attributed to the delicate balance between the van der Waals interactions and polar solvation effects. In the case of *N*,*N*-OME, the van der Waals interactions with the lipid tails are exceptionally strong, exerting a dominant influence and resulting in the deepest free energy minimum. However, these favorable van der Waals interactions are counteracted by the unfavorable solvation energy experienced by *N*,*N*-SER. The presence of a OH group in the indole moiety of *N*,*N*-SER induces strong interactions with bulk water, including hydrogen bonding. These interactions must be disrupted when the ligand permeates the membrane, rendering the reaction less favorable. Consequently, the permeation of *N*,*N*-SER is less favorable than that of the unsubstituted ligand *N*,*N*-TRY, despite the former having more favorable ligand-membrane vdW interactions. Unlike the N,N-methylated ligands, the free-energy minimum of both the N-methylated and unmethylated ligands undergoes a shift in the position with the indole substitution (Figure 7A) because the nonpolar interactions with between the amino group and the membrane tails are less important.

Specifically, the SER ligands exhibit their minimum energy state close to the lipid headgroups, primarily due to interactions between the –OH group and the polar headgroups (*z* = 15.2 Å). In contrast, the OME ligands position their minimum energy state closer to the lipid tails (*z* = 11.2 Å) because of the hydrophobic interactions with the nonpolar lipid tails. At the center of the bilayer, the primary factors influencing the interaction energy are Δ*G*_vdW_ which follows the same trend as the dimethylated compounds, OME > TRY > SER, and Δ*G*_gb_, which is again less favorable for SER due to the high desolvation penalty when the permeation of the membrane occurs.

### Indole –OH Position

**Figure 8:**
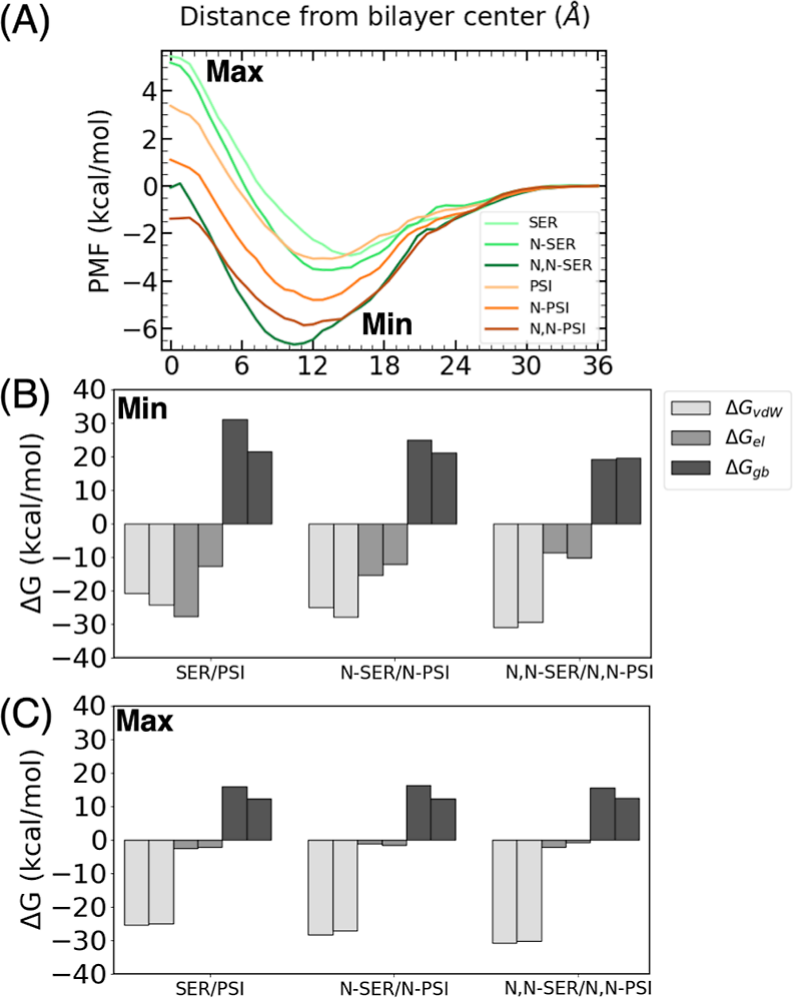
(A) PMF for PSI, *N*-PSI, *N*,*N*-PSI, SER, *N*-SER, and *N*,*N*-SER. Decomposition of the energy into van der Waals (Δ*G*_vdw_), electrostatic (Δ*G*_el_), and implicit generalized Born solvation, (Δ*G*_gb_), in (B) minima and (C) maxima of the compounds.

To assess the impact of the –OH group’s position in the indole moiety, the compounds PSI and SER were compared in all three alkylated forms. One would anticipate a comparable PMF between these two species, considering the similarity; however, contrary to expectations, there are notable differences observed in the profiles (Figure 8A).

All the PSI compounds follow the previously discussed trend of shifting the position of the minima, attributed to the stronger van der Waals interactions with the lipid tails as the methyl substitution progresses from PSI to *N*,*N*-PSI. When comparing the primary amine compounds PSI and SER, the depth of the free energy minimum is similar for both compounds, albeit slightly deeper for PSI. This suggests that the position of the –OH group in the indole ring is not a significant factor influencing the strength of interactions when the amine group is unsubstituted. Regarding the position of the minimum energy state, in both cases, it is situated close to the polar lipid headgroups, as expected due to the polar nature of the OH group. However, for PSI, the minimum is closer to that of the lipid tails.

In the case of the methylated compounds (*N*-PSI vs *N*-SER), the free energy minimum is once again slightly deeper and closer to the lipid tails for *N*-PSI compared to *N*-SER. Notably, due to the methylation of the amine group, both minima have shifted toward the lipid tails in comparison to PSI and SER. The basis for the stronger interactions between PSI and *N*-PSI with the membrane than between SER and *N*-SER with the membrane becomes evident in the MMGBSA decomposition analysis. Specifically, the analysis reveals that the van der Waals interactions are stronger and the polar solvation is less unfavorable for the PSI compounds compared to that of the SER counterparts. These energy terms compensate for the stronger electrostatic interactions found for the SER derivatives than for the PSI ones. The reason for this behavior is that the OH group in SER is more accessible to other molecules than in PSI, and thus the interactions with the polar heads are more important. In the same way, the interactions between SER and the water molecules when the drug is in the bulk solvent are also stronger, making the desolvation process to enter the bilayer more unfavorable in SER than in PSI.

Interestingly, in the case of the double-methylated amine compounds (*N*,*N*-PSI vs *N*,*N*-SER), the situation reverses. The minimum energy state is deeper and closer to the lipid tails for *N*,*N*-SER compared with *N*,*N*-PSI. This shift is attributed to a significant change in the nature of the interactions. As observed in the MMGBSA analysis, electrostatic interactions are now weaker, and the desolvation penalty is very similar for *N*,*N*-PSI than for *N*,*N*-SER. This observation underscores the complexity of drug design as the permeation through the membrane depends on various interconnected factors. As we approach the maxima, there is little variation in the van der Waals and electrostatic contributions, but there is a noticeable increase in the polar solvation for all three SER compounds. The key distinction between these positional isomers lies in their amphipathic nature, with the PSI compounds exhibiting a relatively lower polar character. This characteristic enables them to have a reduced energy barrier to overcome compared with the other compounds.

### Protonation State

**Figure 9:**
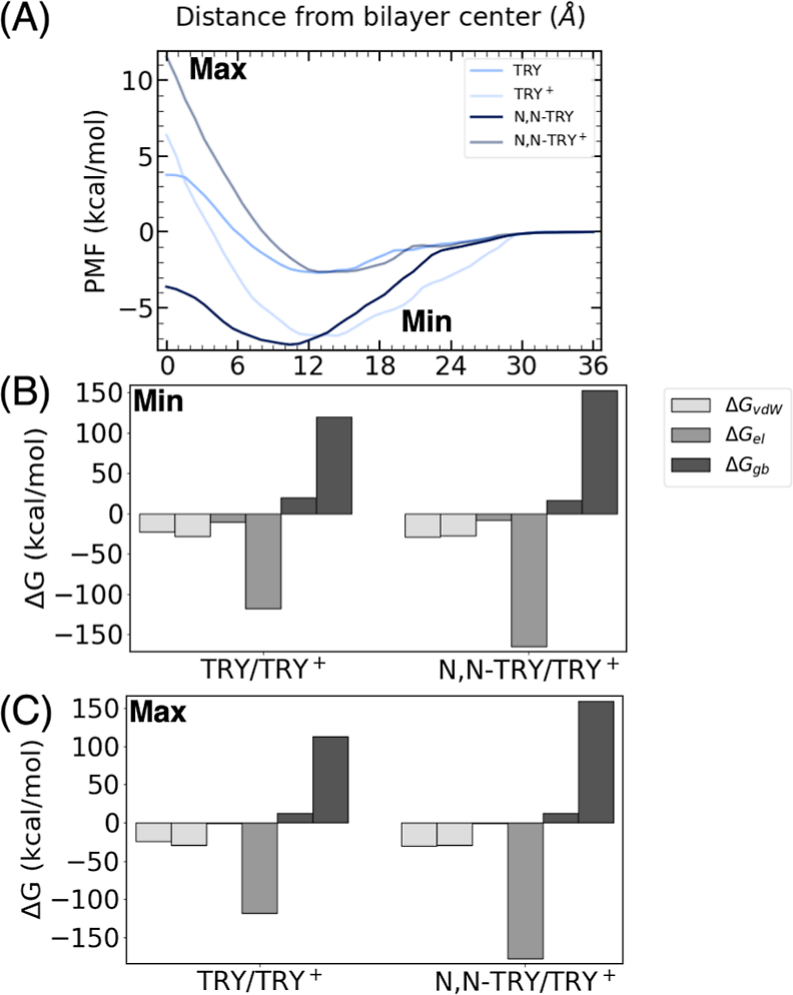
(A) PMF for TRY, *N*,*N*-TRY, TRY^+^, and *N*,*N*-TRY^+^. Decomposition of the energy into van der Waals (Δ*G*_vdw_), electrostatic (Δ*G*_el_), and implicit generalized Born solvation, (Δ*G*_gb_), in the (B) minima and (C) maxima of the compounds.

Assuming a stable physiological pH of 7.4, all of the species under investigation will be mainly found in their ionized state in solution. As shown above, in Table 1, both TRY^+^ and *N*,*N*-TRY^+^ are highly impermeable in the ionized state, which means that those molecules will permeate the membrane in their neutral state and be protonated again in the cytosol. To show that only neutral species can permeate the lipid bilayer, the PMF of TRY and *N*,*N*-TRY was compared with their protonated analogues (Figure 9A). As discussed above, the ligands that are not methylated in the amine group, such as TRY, present their free energy minimum close to those of the polar heads, where the electrostatic interactions dominate. Thus, when TRY is protonated and charged positively (TRY^+^), the electrostatic interactions become stronger (see Figure 9B), and the free energy minimum gets deeper (see Figure 9A). However, since the interactions between a cation and the nonpolar tails are unfavorable, the energy barrier to go through the nonpolar region of the membrane significantly increases when TRY is protonated.

In the case of the *N*,*N*-TRY ligand, the situation is drastically different. Since the free-energy minimum of *N*,*N*-TRY is located close to the tails due to the favorable interactions between the methyl groups and the nonpolar tails, the protonation of the ligand is energetically unfavorable, and the minimum energy drastically increases and is shifted toward the polar heads. For both positively charged ligands, TRY^+^ and *N*,*N*-TRY^+^, the interaction with the polar heads at the minimum of the free energy profile is clearly dominated by the interaction with the phosphate groups. Specifically, the different contributions to the electrostatic interaction between the polar heads and the ligands are the following: choline 20%, phosphate 66%, and glycerol 14% for TRY^+^, while it is choline 24%, phosphate 66%, and glycerol 10% for *N*,*N*-TRY^+^.

Interestingly, the protonated form of TRY displays a deeper minimum compared to the protonated *N*,*N*-TRY, in contrast to the trend observed in their neutral counterparts. It is noteworthy that both minima are positioned approximately the same distance from the center of the bilayer, specifically, at 13.4 Å. The fact that the position of the protonated *N*,*N*-TRY does not shift toward the lipid tails when compared to the protonated TRY, as previously observed for the neutral molecules, signifies that the electrostatics prevail over the van der Waals contribution, as it can be observed in Figure 9B. Indeed, the MMGBSA analysis reveals a striking similarity in the van der Waals contribution between the protonated and neutral forms of TRY. However, a disparity arises in the electrostatic counterpart, which is greater for the protonated TRY. This indicates that the protonation state enhances the electrostatic interactions with the polar heads of the membrane, consequently leading to a more pronounced and deeper energy minimum. A steep barrier is present until reaching the bilayer center at 0 Å. Although the depth of the minima differs, the overall barrier to the center is the same for both protonated molecules, ≈14 kcal/mol. This indicates that these compounds are highly impermeable, as is also evident from the effective permeabilities (log *P*_eff_).

## Conclusions

The investigation of the permeation behavior of psychedelics is crucial to understand the factors that control the entrance to the cells and the subsequent interaction with the 5-HT_2A_R. Such knowledge is of fundamental importance in the design of new compounds with enhanced activity. In this study, by employing classical MD in conjunction with US, we were able to effectively capture and describe the distinct membrane permeation behaviors arising from chemical substitutions in different tryptamine derivatives.

All the compounds permeate from the bulk water phase to the membrane’s head groups and exhibit notable energy minima, situated approximately 14 to 10 Å away from the barrier located at the bilayer’s center. The alkylation of the monoamine induces a notable shift in the energy minima, with the *N*,*N*-compounds exhibiting the closest proximity to the lipid tails. Furthermore, this alkylation process serves to deepen the energy minima and diminishes the barrier at the bilayer’s central region, thus rendering these compounds the most permeable among the tested molecules. In contrast, indole substitution does not induce a shift in the minima but solely deepens them for the N,N-alkylated compounds, signifying that alkylation exerts a greater degree of influence than indole substitution. Conversely, *N*-alkyl and monoamine compounds are more sensitive to indole substitution as they induce both a shift and a deepening of the minima.

Incorporating a methoxy group results in the deepest minima among all of the compounds. The positioning of the OH group demonstrates a negligible impact on the interaction strength when the amine group remains unsubstituted. Intriguingly, a reversal of trends becomes apparent when comparing the *N*,*N*-PSI and *N*,*N*-SER compounds. In this context, the *N*,*N*-SER compound exhibits a deeper minimum, which is explained by the weaker van der Waals interactions and higher desolvation penalty experienced by *N*,*N*-PSI. At the maxima, the height primarily hinges upon van der Waals interactions, thus affording N,N-alkylated compounds superiority over mono- and unsubstituted counterparts. Furthermore, the –OCH3 substitution significantly diminishes the barrier height, in contrast to the –OH group, highlighting the marked impact of these structural modifications on barrier energetics. Since monoamines position their free energy minimum close to the polar lipid heads, the protonated analogues experience significantly higher electrostatic interactions, inducing a deeper minimum. In contrast, for *N*,*N*-TRY^+^, the minimum does not shift toward the lipid tails compared to the TRY^+^, as was observed in the neutral counterparts, resulting in unfavorable electrostatic interactions compared to TRY^+^, and consequently a shallower minimum. A steep, energetically equivalent barrier is present for both, rendering these protonated compounds highly impermeable.

It is important to mention that one limitation of the simulations performed here is the lack of a description of the concentration gradient due to the ion trapping effect, which could modify the dynamics of the membrane transport. In addition, it would be desirable to have more experimental data to compare in a more exhaustive way the conclusions extracted by our calculations. Despite these limitations, our study demonstrates the application of state-of-the-art computational techniques for the comprehensive characterization of the physicochemical properties of tryptamines underlying compound-specific membrane permeabilities. Our results are in agreement with the available experimental data and provide novel insights into the molecular profile of classic psychedelics and related therapeutic compounds, which have significant implications for drug development.

## Supporting Information Available

Analysis of membrane equilibration with plots of area per lipid vs time and electron density vs distance from the bilayer center and analysis of potential of mean force convergence including probability distribution and PMF data (PDF)
