# Supplementary material for: Membrane Permeation of Psychedelic Tryptamines by Dynamic Simulations
Source: Biochemistry. 2024 Feb 7;63(4):419–28. doi: 10.1021/acs.biochem.3c00598 (PMC10882957; doi:10.1021/acs.biochem.3c00598)
Supplement: Supplementary file 1 — bi3c00598_si_001.pdf [file bi3c00598_si_001.pdf]

Supporting Information for Membrane Permeation of Psychedelic Tryptamines by Dynamic Simulations

Vito F. Palmisano,[†,‡] Claudio Agnorelli,[¶,§] Andrea Fagiolini,[§] David Erritzoe,[¶] David Nutt,[¶] Shirin Faraji,[*,‡] and Juan J. Nogueira[*,†,‖]

†*Department of Chemistry, Universidad Autonoma de Madrid, Madrid, Spain.*

‡*Theoretical Chemistry Group, Zernike Institute for Advanced Materials, University of Groningen, Groningen, The Netherlands.*

¶*Center for Psychedelic Research, Division of Psychiatry, Department of Brain Science, Imperial College of London, London, United Kingdom*

§*Unit of Psychiatry, Department of Molecular Medicine, University of Siena, Siena, Italy*

‖*IADCHEM, Institute for Advanced Research in Chemistry, Universidad Autonoma de Madrid, Madrid, Spain*

E-mail: s.s.faraji@rug.nl; juan.nogueira@uam.es

Membrane Equilibration

As described above, initially, a 200 ns of MD simulation was performed to achieve structural equilibration of the solvated POPC membrane. Two properties were calculated to evaluate the convergence of the equilibration process. The first property to be computed was the area per lipid, defined as the average area that a single lipid molecule occupies on the interface. Figure 1A shows that after around 20 ns, the area per lipid oscillates around a value of

64.4Å^2, which is in good agreement with the experimental result of 64.3Å^2 at 303 K.[1]

The second property was the electron density along the z-axis of the full bilayer. The density profile analysis (Figure 1B) reveals two main peaks approximately at -18 Å and 18 Å from the center of the bilayer, corresponding to the phosphate groups' locations within the POPC membrane. Moving towards the bilayer center, the electron density gradually diminishes, reaching its minimum at the terminal region of the lipid tails, farthest from the polar heads. The electron densities calculated for the first 20 ns and the entire 200 ns of the simulation are very similar, indicating that there are no significant diffusion processes occurring within the lipid bilayer. Both properties suggest that the bilayer is well equilibrated and stable throughout the simulation of 200 ns.

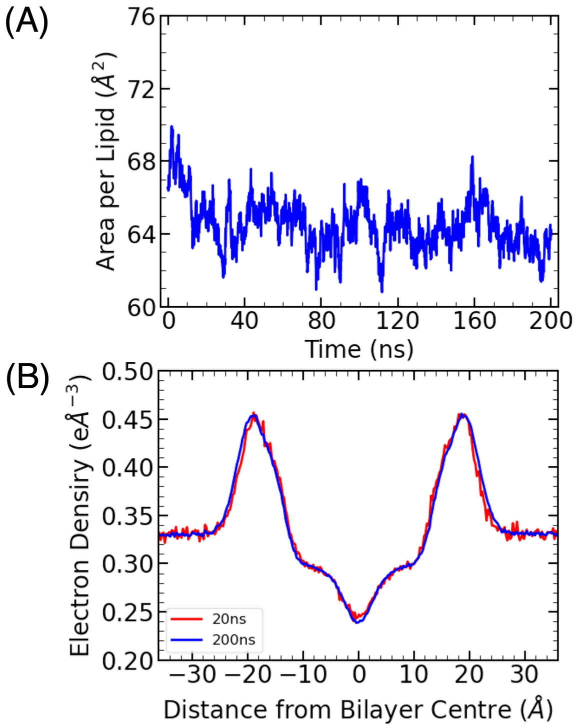

Figure 1: (A) Area per lipid in 200 ns of membrane equilibration. (B) Electron density profile through the membrane for 20 and 200 ns of equilibration.

Potential of Mean Force Convergence

Two important factors that determine the convergence of the PMF are the number of windows and the simulation time per window. Figure 2A shows the probability distribution of the reaction coordinate for all the windows, for the largest compounds in size (N,N-OME) taken as an example. Each distribution overlaps well with the two neighboring distributions, a requirement which is needed to obtain accurate free energy profiles. Furthermore, each maximum aligns approximately with the chosen value of the applied harmonic restraint. This indicates that the restraint's strength is sufficient to keep the ligand in the desired position inside the membrane, preventing excessive diffusion, while still allowing for a reasonably broad probability distribution.

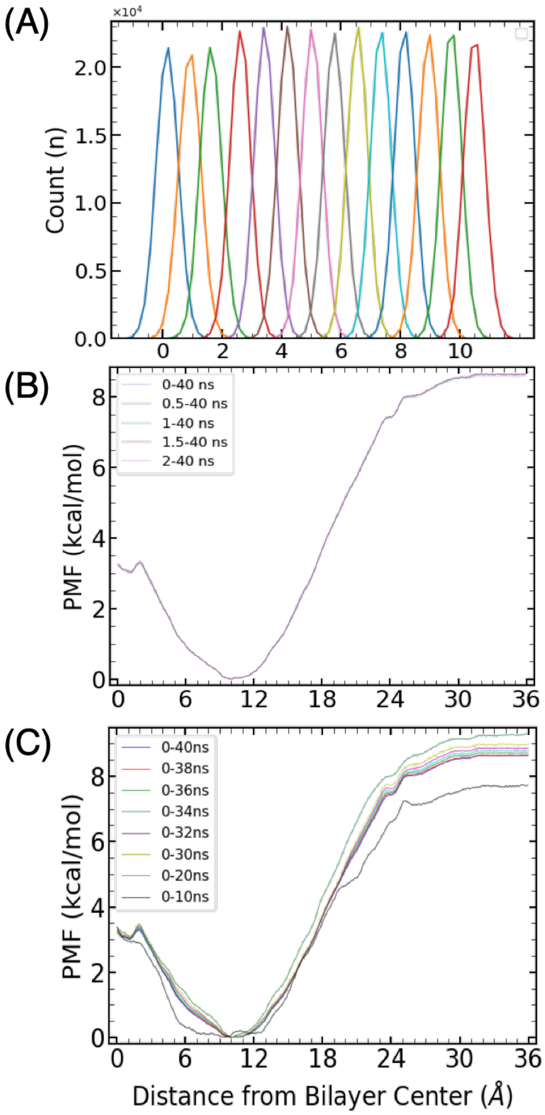

Figure 2: (A) Probability distribution of the reaction coordinate for TRY across the 45 windows used for umbrella sampling. (B) Potential of mean force (PMF) computed by removing 0.5, 1, 1.5 and 2 ns from the beginning of each window. (C) PMF computed by removing 2, 4, 6, 8, 10, 20 and 30 ns from the end of each window.

Figure 2B displays the PMF computed for the full simulation time (40 ns per window) and for different time intervals, excluding the initial steps of each window. Specifically, time intervals of 0.5 ns, 1 ns, 1.5 ns, and 2 ns were excluded. No significant changes are observed along the minima and barriers of the PMF, indicating that no initial portions of the simulations need to be discarded. To ensure convergence of the simulation time, a similar

procedure was followed, but this time excluding the final steps of each window (Figure 2C). Specifically, the last 2, 4, 6, 8, 10, 20, and 30 ns were removed, which means that the PMF was computed by considering the first 38, 36, 34, 32, 30, 20 and 10 ns of each window. When examining the different profiles one can conclude that convergence is achieved after 30 ns of simulations time per window. Both convergence analyses were performed on the largest molecule, N,N-OME. Having achieved fully converged profiles, the subsequent section will present an analysis of the main features of the permeation profiles. This will be followed by an analysis of the influence of amine alkylation, ring substitution, ring position and protonation on the PMF and permeation for all the compounds under investigation.

References

(1) Kučerka, N.; Nieh, M.-P.; Katsaras, J. Fluid phase lipid areas and bilayer thicknesses of commonly used phosphatidylcholines as a function of temperature. *Biochimica et Biophysica Acta (BBA)-Biomembranes* **2011**, *1808*, 2761–2771.
